# Enhanced Object Detection in Thangka Images Using Gabor, Wavelet, and Color Feature Fusion

**DOI:** 10.3390/s25113565

**Published:** 2025-06-05

**Authors:** Yukai Xian, Yurui Lee, Te Shen, Ping Lan, Qijun Zhao, Liang Yan

**Affiliations:** 1 School of Information Science and Technology, Tibet University, Lhasa 850000, China; yukai@stu.utibet.edu.cn (Y.X.);; 2 School of Humanities, Tibet University, Lhasa 850000, China; li_yu_rui@163.com (Y.L.);; 3 College of Computer Science, Sichuan University, Chengdu 610065, China

**Keywords:** Thangka detection, wavelet transform, prior-guided attention, multi-scale object recognition

## Abstract

Thangka image detection poses unique challenges due to complex iconography, densely packed small-scale elements, and stylized color–texture compositions. Existing detectors often struggle to capture both global structures and local details and rarely leverage domain-specific visual priors. To address this, we propose a frequency- and prior-enhanced detection framework based on YOLOv11, specifically tailored for Thangka images. We introduce a Learnable Lifting Wavelet Block (LLWB) to decompose features into low- and high-frequency components, while LLWB_Down and LLWB_Up enable frequency-guided multi-scale fusion. To incorporate chromatic and directional cues, we design a Color-Gabor Block (CGBlock), a dual-branch attention module based on HSV histograms and Gabor responses, and embed it via the Color-Gabor Cross Gate (C2CG) residual fusion module. Furthermore, we redesign all detection heads with decoupled branches and introduce center-ness prediction, alongside an additional shallow detection head to improve recall for ultra-small targets. Extensive experiments on a curated Thangka dataset demonstrate that our model achieves 89.5% mAP@0.5, 59.4% mAP@[0.5:0.95], and 84.7% recall, surpassing all baseline detectors while maintaining a compact size of 20.9 M parameters. Ablation studies validate the individual and synergistic contributions of each proposed component. Our method provides a robust and interpretable solution for fine-grained object detection in complex heritage images.

## 1. Introduction

Thangka is a traditional form of Tibetan Buddhist art that features intricate details, symbolic iconography, and vibrant color compositions. Due to its rich visual complexity and cultural significance, automated analysis of Thangka imagery has become an important research direction in digital heritage preservation.

Object detection has evolved remarkably over the past decade, from two-stage methods such as Faster R-CNN [1]—which introduced region proposal networks and established the two-stage paradigm—to single-stage detectors like SSD [2] and YOLO [3], which improve efficiency by predicting bounding boxes and classes in a single pass. Advances including SSD’s multi-scale feature maps and default boxes, YOLO’s regression-based pipeline, RetinaNet’s focal loss for addressing foreground–background imbalance [4], and Cascade R-CNN’s multi-stage IoU refinement [5] have significantly increased detection accuracy and speed. EfficientDet [6] further reduced computational cost with compound scaling and BiFPN fusion, while anchor-free detectors like FCOS [7] simplified pipelines by removing anchor boxes. Recently, DETR [8] reframed detection as a set prediction task using transformers, enabling end-to-end optimization without hand-crafted components such as NMS. These classical algorithms provide the foundation for task-specific frameworks. Our method builds on these and adapts them to Thangka image analysis, where classical architectures often fail to fully exploit domain-specific priors like wavelet structures, color decomposition, and orientation sensitivity.

In recent years, the application of computer vision to Thangka digital preservation has grown rapidly, giving rise to algorithms tailored for its unique textures, dense iconography, and stylistic variance. Early works explored classification and feature analysis with dual-branch contrastive learning and texture-aware attention [9], geometric analysis of facial features [10], and CBIR with LBP, HSV, and hashing for authentication [11]. Segmentation studies proposed networks with atrous convolutions and cross-layer fusion [12] or weakly supervised polygon masks [13]. Restoration and inpainting leveraged wavelet- and structure-guided methods [14] and diffusion-based text-edge frameworks [15]. Multimodal research introduced transformer-based image–text alignment [16], semantic-prompted captioning [17], and sketch-based colorization with instance-normalized fusion [18]. Detection in Thangka remains challenging due to dense, small-scale objects: few-shot deformable convolution methods [19], YOLOv5 with attention [20], and YOLOv8 with attention fusion [21] all report progress. Further, hyperspectral imaging for super-resolution [22] and restoration methods [14,15] reflect the diversity of approaches. Collectively, these studies advance the classification, detection, segmentation, restoration, and multimodal modeling of Thangka art [9,10,11,12,13,14,15,16,17,18,19,20,21,22].

Despite these advances, object detection on Thangka images is still under-explored and remains challenging. Existing detectors have three main limitations. First, they do not include explicit frequency decomposition mechanisms. As a result, they cannot effectively model both large color regions and fine edge structures that are common in Thangka iconography. Second, standard upsampling methods in the neck often introduce aliasing artifacts. This weakens semantic alignment across scales, especially for small and dense elements. Third, most detection pipelines do not use perceptual priors such as color saliency and directional texture. These priors are important for guiding attention within Thangka compositions.

To address these issues, we propose a detection framework based on YOLOv11 and tailored for Thangka images. Our architecture features a Learnable Lifting Wavelet Block (LLWB) for frequency decomposition; LLWB_Down and LLWB_Up modules for frequency-guided multi-scale fusion; a dual-branch Color-Gabor Block (CGBlock) with HSV and Gabor attention, combined via the Color-Gabor Cross Gate (C2CG); and redesigned detection heads with an additional shallow head for ultra-small, dense targets.

Extensive experiments on a curated Thangka detection dataset show our method outperforms classical and transformer-based detectors in mAP@0.5, mAP@[0.5:0.95], and recall, while maintaining compact model size. Ablation studies further validate that each proposed module contributes to overall performance. By incorporating frequency modeling, domain-specific priors, and scale-sensitive heads, our framework provides a robust and interpretable solution for Thangka object detection.

## 2. Related Work

### 2.1. YOLOv11 Architecture

**Figure 1:**
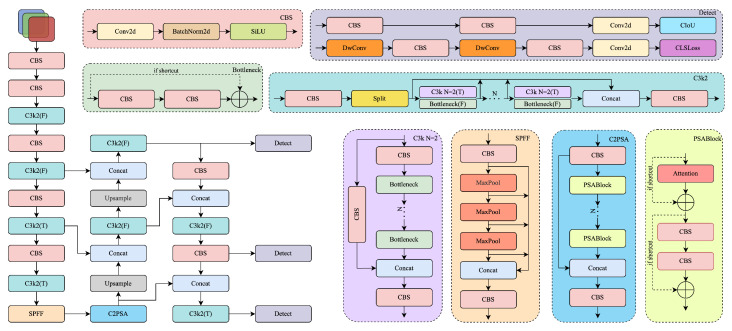
Overall architecture of YOLOv11, consisting of a lightweight backbone, spatial attention-enhanced neck, and modular prediction head [23].

YOLOv11, introduced by Ultralytics in 2024 [23], achieves an improved balance between speed and accuracy for real-time object detection through architectural updates across the backbone, neck, and prediction head. Figure 1 shows the main changes in the YOLOv11 backbone. The backbone replaces the older C2f block with a lightweight C3k2 module, reducing computation while maintaining robust feature representation. The SPPF module is retained for multi-scale context, and C2PSA spatial attention helps the model focus on dense regions.

The neck employs multi-path feature fusion and C3k2 modules to improve cross-scale aggregation, with additional attention mechanisms preserving semantic detail. The prediction head includes modular detection branches built from CBS (Conv–BN–SiLU) blocks and a unified Detect layer, facilitating accurate detection for both large and small objects. Overall, YOLOv11 integrates spatial attention and backbone refinements to achieve strong results on benchmarks like COCO. In this work, we use YOLOv11 as our base detector and further enhance it with frequency-aware modeling and domain-specific visual priors for the unique challenges of Thangka object detection.

### 2.2. Wavelet-Based Multi-Resolution Feature Extraction

Wavelet transforms are valued in object detection for their multi-resolution analysis and ability to preserve spatial and frequency information. Early approaches such as the Quaternionic Wavelet Transform (QWT) [24] and WBCT with saliency mapping [25] enhanced local feature extraction and robustness to cluttered backgrounds. The integration of wavelet transforms into CNNs has become more advanced, as seen in WCNN3D [26], which uses DWT/IWT for lightweight representation and WTConv layers [27] that provide large receptive fields and noise robustness. In YOLO-based designs, DI-YOLOv5 [28] incorporates dual-wavelet convolution modules (DWCM) to preserve the features of small objects and models spatial dependencies using CSPCoA. This progression in frequency-aware architectures—demonstrated to generalize well across vision models [27]—inspires us to introduce frequency-aware modules into our detection framework to better capture fine-grained details in complex Thangka imagery.

### 2.3. Color-Guided Modeling in Object Detection

Color information is a crucial visual cue for object detection. Early methods leveraged statistical modeling of spatial color layouts [29], compact color attributes [30], and color name spaces [31] to capture chromatic contrast and salient objects robustly. With deep learning, color priors are increasingly integrated into neural networks: cognitively driven attention mechanisms [32] fuse color with CNN features to guide detection, while channel analysis [33] shows blue channel emphasis benefits medical imaging. In our approach, we adopt color-guided modules to adaptively weight regions by chromatic concentration, which is particularly effective for highlighting figures within the colorful and complex backgrounds of Thangka images.

### 2.4. Gabor-Based Feature Modeling for Texture and Orientation Analysis

Gabor filters have long been valued for their spatial-frequency and orientation selectivity in vision tasks. Deep learning integration began with steerable Gabor filters in convolutional layers, improving robustness to scale and rotation with compact models [34]. Gabor-based methods have been applied to saliency detection [35], small-sample object detection with RPNs and feature pyramids [36], and, via AGCNs [37], learnable convolutional kernels for improved generalization under spatial distortions. Gabor analysis has also extended into remote sensing, with fractional Gabor spectrum methods enhancing SAR target detection [38]. Building on these advances, we integrate Gabor-based modules to capture the texture and orientation-sensitive features characteristic of Thangka art.

## 3. Methods

YOLOv11 strikes an appealing balance between accuracy and speed through its compact CBS backbone, the C3k2 (F/T) blocks, the SPFF spatial–prior fusion, and the detail-aware C2PSA module.

Nevertheless, when applied directly to Thangka imagery, three limitations emerge. First, the purely spatial backbone struggles to simultaneously capture large chromatic regions and fine-grained edge details, due to the lack of explicit low-/high-frequency modeling. Second, conventional upsampling in the neck introduces aliasing, making cross-scale semantic alignment unstable. Third, the network exploits neither chromatic saliency nor directional texture priors, leading to sub-optimal recall on tiny ornaments and densely packed details.

**Figure 2:**
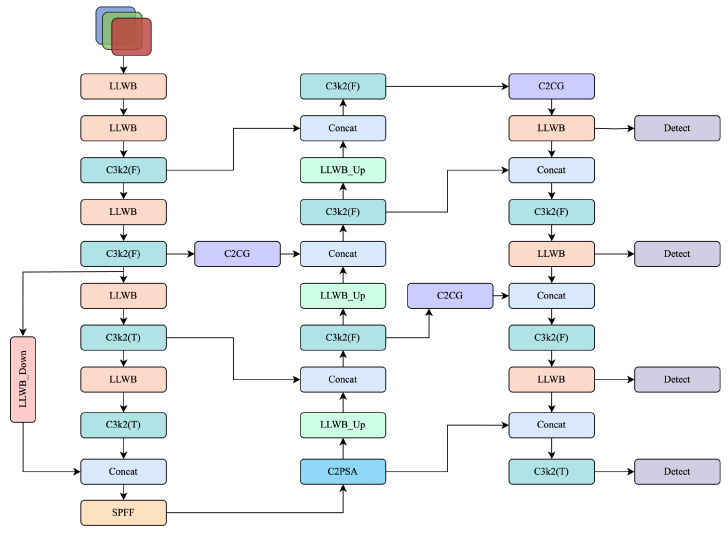
Overview of the proposed architecture. The design is based on YOLOv11, enhanced by frequency decomposition modules (LLWB, LLWB_Down, LLWB_Up), C2CG, and an additional shallow detection head.

To overcome these issues, we introduce a frequency- and prior-aware enhancement on top of YOLOv11. As shown in Figure 2, our proposed architecture builds upon YOLOv11 by integrating frequency-domain modeling, prior-guided attention, and a shallow detection head. Specifically, all stand-alone convolutional layers are replaced by an LLWB, which decomposes features into low- and high-frequency sub-bands for structural enhancement. An LLWB_Down module is inserted at the end of the backbone, while upsampling operations in the neck are substituted with LLWB_Up, enabling frequency-guided inter-level fusion. In parallel, we design a CGBlock that combines global color histograms with multi-orientation Gabor responses; building on it, a C2CG module is embedded at three key fusion stages to emphasise chromatic and directional cues. Finally, all detection heads are redesigned with a deeper and more modular structure, and an additional shallow head is introduced to enhance recall on ultra-small and densely distributed targets. With only a marginal computational overhead, the resulting network markedly improves robustness and accuracy on the color-rich, fine-grained Thangka detection task.

### 3.1. Wavelet-Based Frequency Modeling

Thangka images exhibit abundant structured patterns, dominant color blocks, and repeated ornamental textures across multiple scales. Convolutional networks, while effective in spatial encoding, suffer from limited receptive fields and lack explicit frequency decomposition capabilities. This restricts their ability to capture global structure and local edge variations simultaneously. In contrast, wavelet transforms provide a joint space–frequency representation by decomposing the input into low-frequency structure and high-frequency detail components. Unlike Fourier transforms, which lose spatial locality, wavelets support multiscale, localized, and direction-sensitive modeling, making them particularly suitable for complex artistic images.

Traditional Discrete Wavelet Transforms (DWTs), however, rely on fixed filters such as Haar or Daubechies, which are not adaptive and cannot be trained end-to-end. To overcome this, we introduce a learnable wavelet transformation based on the lifting scheme, enabling the decomposition filters to be optimized during training. We design three wavelet-based modules: LLWB, LLWB_Down, and LLWB_Up. These modules integrate frequency modeling into the backbone encoding and the inter-level feature fusion process. In addition, we propose a Cross-Band Channel Attention (CBCA) mechanism that adaptively reweights wavelet subbands to emphasize structurally informative components. This design enhances the network’s capacity to encode both global semantic structure and fine-grained edge cues in Thangka detection.

#### Learnable Wavelet Modules and Cross-Scale Frequency Modeling

**Figure 3:**
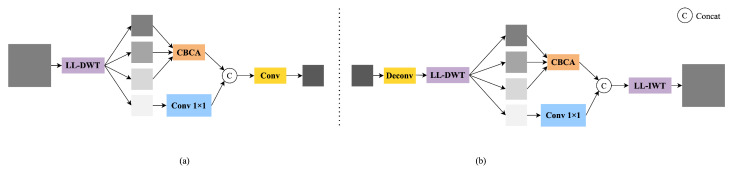
Detailed structure of our frequency-aware modules. (**a**) The LLWB_Down module extracts frequency residuals from backbone features. It performs LL-DWT to obtain four subbands, applies CBCA to the low-frequency groups, and compresses the output via 1×1 and 3×3 convolutions. (**b**) The LLWB_Up module reconstructs upsampled features with improved structural fidelity. It first upsamples and applies LL-DWT, followed by CBCA and LL-IWT for inverse transformation. These modules enhance cross-scale consistency while preserving fine-grained frequency detail.

To enhance the network’s ability to model multi-scale structures, we introduce a complete frequency modeling path that spans both the backbone and neck stages. We implement this by replacing standard convolutions with learnable lifting wavelet transforms (LL-DWT and LL-IWT). We also design two cross-level frequency-guided modules, LLWB_Down and LLWB_Up, and supplement them with CBCA for subband-specific enhancement. The overall structures of LLWB_Down and LLWB_Up are shown in Figure 3.

Unlike traditional discrete wavelet transforms that use fixed filters (e.g., Haar, Daubechies), our lifting-based formulation enables the decomposition operators to be learned from data. For a 1D input signal $\left\{x_{2i},x_{2i+1}\right\}$, the lifting-based forward transform can be expressed as:

(1) $$d_{i}=x_{2i+1}-P_{\theta }\left(x_{2i}\right),\;s_{i}=x_{2i}+U_{\phi }\left(d_{i}\right),$$

where $P_{\theta }\left(\cdot \right)$ and $U_{\phi }\left(\cdot \right)$ are trainable prediction and update functions implemented as 1×1 convolutions. Applying this scheme along both spatial dimensions yields four subbands: $I_{LL}$ (low-frequency structure) and $I_{LH}$, $I_{HL}$, $I_{HH}$ (high-frequency directional details). The inverse lifting transform is defined symmetrically as:

(2) $${\hat{x}}_{2i}=s_{i}-U_{\phi }\left(d_{i}\right),\;{\hat{x}}_{2i+1}=d_{i}+P_{\theta }\left({\hat{x}}_{2i}\right),$$

allowing full reconstruction of the original spatial feature. In contrast to fixed wavelets, our LL-DWT and LL-IWT are differentiable and trainable, enabling adaptive structural modeling tailored to Thangka images.

Based on these operators, we design the LLWB_Down module at the end of the backbone to extract structural frequency residuals. Given an input feature map *F*, we first apply LL-DWT:

(3) $$\left\{I_{LL},I_{LH},I_{HL},I_{HH}\right\}=\mathrm{LL}-\mathrm{DWT}\left(F\right).$$

To enhance the structural representation of decomposed subbands, we design a CBCA mechanism. Unlike conventional channel attention that relies on global pooling and fully connected layers, CBCA directly operates on the spatial and frequency domains to preserve fine-grained locality.

Given three input subbands, $I_{LH}$ (horizontal details), $I_{HL}$ (vertical details), and $I_{LL}$ (low-frequency structure), CBCA proceeds as follows. First, the two directional subbands are fused by element-wise addition:

(4) $$S=I_{LH}+I_{HL}.$$

Next, the fused map *S* is element-wise multiplied with the low-frequency map $I_{LL}$ to generate a structural attention map:

(5) $$A=S⊙I_{LL},$$

where ⊙ denotes element-wise multiplication between feature maps.

Finally, the attention map *A* is added back to $I_{LL}$ via element-wise addition to obtain the enhanced output:

(6) $$O=A+I_{LL}.$$

Formally, we define the CBCA operation as:

(7) $$\mathrm{CBCA}\left(I_{LL},I_{LH},I_{HL}\right)=\left(I_{LH}+I_{HL}\right)⊙I_{LL}+I_{LL}.$$

This design enables CBCA to adaptively highlight informative structural cues by combining directional detail responses with global low-frequency contexts, thereby improving feature discrimination for downstream detection tasks.

**Figure 4:**
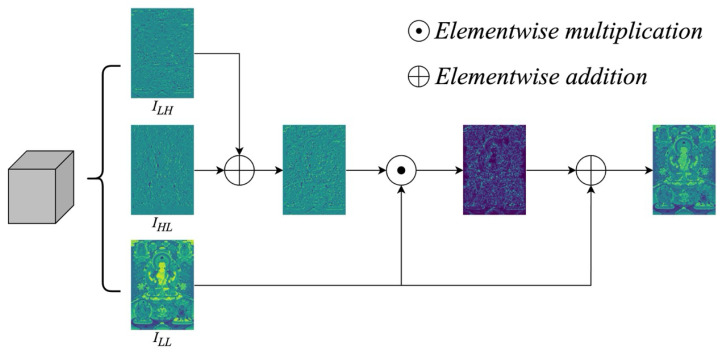
Visualization of the CBCA mechanism. CBCA takes four wavelet subbands from LL-DWT: $I_{LL}$ (low-frequency), $I_{LH}$ (horizontal), $I_{HL}$ (vertical), and $I_{HH}$ (diagonal). It first fuses the directional subbands ($I_{LH}$ and $I_{HL}$) to create a structural response map. This map is reweighted by channel attention and then summed with $I_{LL}$. The result highlights both global structure and fine edge details.

The enhanced low-frequency representation obtained from CBCA (see Figure 4) is concatenated with the transformed high-frequency component, and the combined feature is then compressed through a 3×3 convolution:

(8) $$F_{\mathrm{down}}={\mathrm{Conv}}_{3\times 3}\left(\mathrm{Concat}\left(\mathrm{CBCA}\left(I_{LL},I_{LH},I_{HL}\right),{\mathrm{Conv}}_{1\times 1}\left(I_{HH}\right)\right)\right).$$

Here, Concat(·) denotes channel-wise concatenation, ${\mathrm{Conv}}_{1\times 1}$ denotes a 1×1 convolutional layer applied to $I_{HH}$, and ${\mathrm{Conv}}_{3\times 3}$ denotes a 3×3 convolution used to compress and fuse the concatenated features. This frequency-guided feature is passed from the backbone to the neck as a residual structural prior.

In the neck, we replace conventional upsampling operations with LLWB_Up. As shown in Figure 3, the input feature map is first upsampled via deconvolution, producing $F_{↑}$. This upsampled feature is then passed through an LL-DWT operation to generate four subbands:

(9) $$\left\{J_{LL},J_{LH},J_{HL},J_{HH}\right\}=\mathrm{LL}-\mathrm{DWT}\left(F_{↑}\right).$$

Next, CBCA is applied to the first three subbands to enhance the structural information:

(10) $$J_{\mathrm{CBCA}}=\mathrm{CBCA}\left(J_{LL},J_{LH},J_{HL}\right).$$

Meanwhile, the diagonal detail component $J_{HH}$ is processed through a 1×1 convolution:

(11) $$J_{\mathrm{HH}}^{\prime }={\mathrm{Conv}}_{1\times 1}\left(J_{HH}\right).$$

The resulting features are concatenated along the channel dimension and passed through an inverse lifting wavelet transform:

(12) $$F_{\mathrm{up}}=\mathrm{LL}-\mathrm{IWT}\left(\mathrm{Concat}\left(J_{\mathrm{CBCA}},J_{\mathrm{HH}}^{\prime }\right)\right).$$

Here, Concat(·) denotes channel-wise concatenation. This frequency-guided reconstruction enhances cross-scale consistency and restores fine structural details that are often lost in conventional upsampling.

This reconstruction process aligns multi-scale semantic features in the frequency domain and mitigates aliasing artifacts introduced by naive interpolation, thereby improving fine-grained structure recovery and robustness in detecting complex targets.

### 3.2. Wavelet-Enhanced Convolutional Module

To improve the frequency representation capability during feature extraction, we design a LLWB to replace all standalone convolutional layers in YOLOv11. This module introduces hierarchical decomposition, cross-frequency attention enhancement, and dual-path reconstruction to better model structural and textural information.

**Figure 5:**
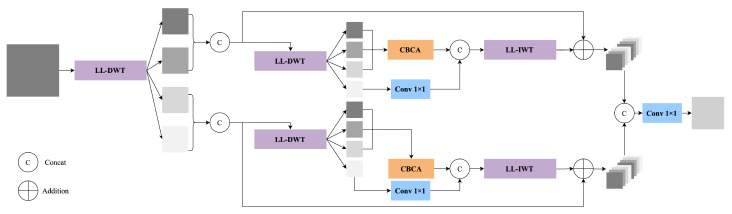
Illustration of the LLWB. The input feature is first decomposed into four frequency subbands using LL-DWT. Each subband is processed via two parallel LLWB_Down branches, where the low-frequency components are enhanced by the CBCA mechanism and concatenated with the projected high-frequency part. The fused representations are then reconstructed via LLWB_Up modules using LL-IWT, producing refined feature maps that preserve both structural integrity and high-frequency texture. The two reconstructed paths are fused and projected to form the final output.

As shown in Figure 5, Given an input feature map $F\in R^{C\times H\times W}$, we first apply LL-DWT to decompose it into four subbands:

(13) $$\left\{I_{LL},I_{LH},I_{HL},I_{HH}\right\}=\mathrm{LL}-\mathrm{DWT}\left(F\right),$$

where $I_{LL}$ captures low-frequency structure, while $I_{LH},I_{HL},I_{HH}$ represent directional high-frequency details.

The four subbands are processed along two symmetric paths. In the first path, $I_{LL}$ and $I_{HH}$ are concatenated and passed through another LL-DWT:

(14) $$\left\{I_{LL}^{\prime },I_{LH}^{\prime },I_{HL}^{\prime },I_{HH}^{\prime }\right\}=\mathrm{LL}-\mathrm{DWT}\left(\mathrm{Concat}\left(I_{LL},I_{HH}\right)\right).$$

The low-frequency subbands $I_{LL}^{\prime },I_{LH}^{\prime },I_{HL}^{\prime }$ are enhanced by a CBCA module:

(15) $$S_{\mathrm{CBCA}}=\mathrm{CBCA}\left(I_{LL}^{\prime },I_{LH}^{\prime },I_{HL}^{\prime }\right),$$

while the high-frequency subband $I_{HH}^{\prime }$ is projected through a 1×1 convolution:

(16) $$S_{HH}^{\prime }={\mathrm{Conv}}_{1\times 1}\left(I_{HH}^{\prime }\right).$$

The outputs $S_{\mathrm{CBCA}}$ and $S_{HH}^{\prime }$ are concatenated and passed through LL-IWT to reconstruct the feature map:

(17) $$F_{\mathrm{recon}1}=\mathrm{LL}-\mathrm{IWT}\left(\mathrm{Concat}\left(S_{\mathrm{CBCA}},S_{HH}^{\prime }\right)\right).$$

This reconstructed feature is added element-wise to the original concatenated feature $\mathrm{Concat}(I_{LL},I_{HH})$ to form the output of the first path:

(18) $$F_{\mathrm{out}1}=F_{\mathrm{recon}1}+\mathrm{Concat}\left(I_{LL},I_{HH}\right).$$

Similarly, in the second path, $I_{LH}$ and $I_{HL}$ are concatenated and processed through LL-DWT:

(19) $$\left\{I_{LL}^{\prime \prime },I_{LH}^{\prime \prime },I_{HL}^{\prime \prime },I_{HH}^{\prime \prime }\right\}=\mathrm{LL}-\mathrm{DWT}\left(\mathrm{Concat}\left(I_{LH},I_{HL}\right)\right).$$

The subbands $I_{LL}^{\prime \prime },I_{LH}^{\prime \prime },I_{HL}^{\prime \prime }$ are enhanced via CBCA:

(20) $$S_{\mathrm{CBCA}}^{\prime \prime }=\mathrm{CBCA}\left(I_{LL}^{\prime \prime },I_{LH}^{\prime \prime },I_{HL}^{\prime \prime }\right),$$

and $I_{HH}^{\prime \prime }$ is passed through a 1×1 convolution:

(21) $$S_{HH}^{\prime \prime }={\mathrm{Conv}}_{1\times 1}\left(I_{HH}^{\prime \prime }\right).$$

The concatenated outputs are reconstructed by LL-IWT:

(22) $$F_{\mathrm{recon}2}=\mathrm{LL}-\mathrm{IWT}\left(\mathrm{Concat}\left(S_{\mathrm{CBCA}}^{\prime \prime },S_{HH}^{\prime \prime }\right)\right),$$

and added to the original concatenation:

(23) $$F_{\mathrm{out}2}=F_{\mathrm{recon}2}+\mathrm{Concat}\left(I_{LH},I_{HL}\right).$$

Finally, the outputs from the two paths $F_{\mathrm{out}1}$ and $F_{\mathrm{out}2}$ are concatenated and compressed via a 1×1 convolution to form the final output:

(24) $$F_{\mathrm{final}}={\mathrm{Conv}}_{1\times 1}\left(\mathrm{Concat}\left(F_{\mathrm{out}1},F_{\mathrm{out}2}\right)\right).$$

This dual-path frequency decomposition and cross-band enhancement design enables LLWB to effectively capture hierarchical structures, directional textures, and fine-grained details, providing a robust foundation for detecting complex elements in Thangka imagery.

### 3.3. Color and Texture Prior-Guided Attention

Thangka images often display clear regional color distributions. Common examples include golden ornaments, red robes, and deep blue backgrounds. In addition, these images contain intricate and directional textures, such as flame outlines, petal structures, and decorative strokes. These characteristics are visually salient and semantically meaningful, yet conventional attention modules often rely solely on deep features, lacking explicit mechanisms to utilize color or directional priors.

**Figure 6:**
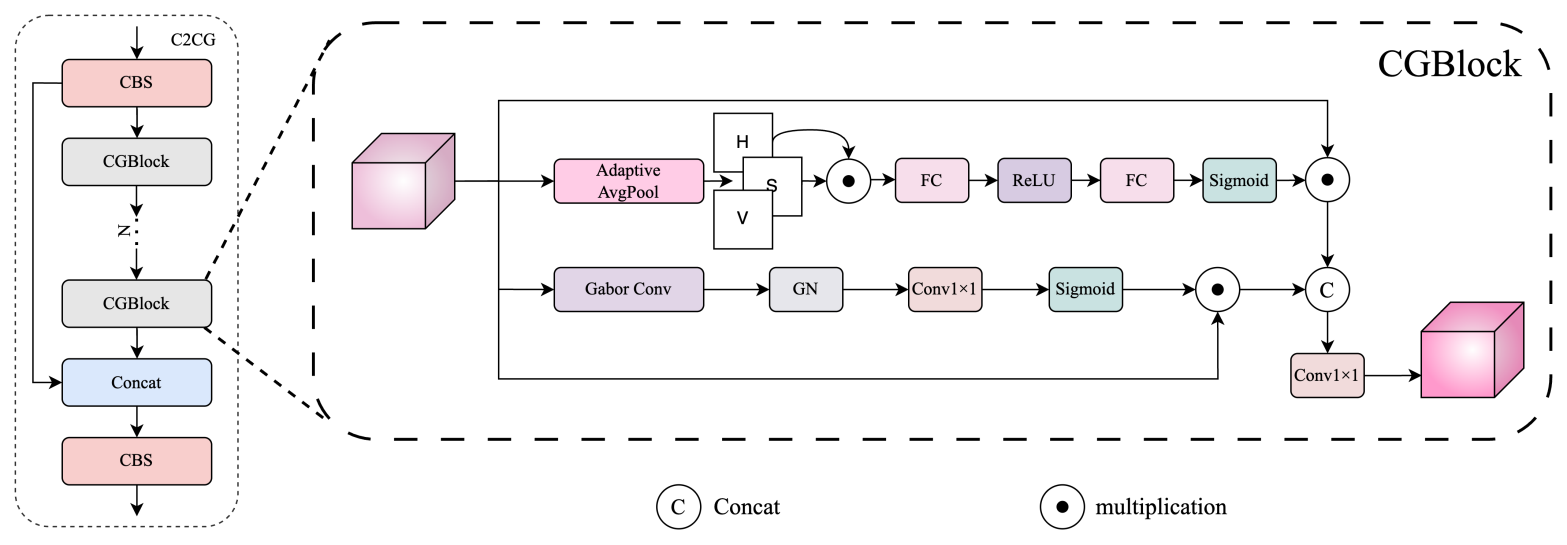
Architecture of the C2CG module. (**Left**) The C2CG structure follows the layout of YOLOv11’s C2PSA module but replaces the original PSABlock with our proposed CGBlock to incorporate color and texture priors. (**Right**) The CGBlock design includes two parallel attention branches: a color-aware branch guided by global HSV statistics, where “H”, “S”, and “V” denote the hue, saturation, and value channels respectively, and a texture-aware branch that aggregates directional responses from multi-orientation Gabor filters. The two attention paths modulate the input feature map and are fused to emphasize perceptually salient and structurally aligned regions.

To address this, we propose a dual-prior attention module named CGBlock, which jointly leverages global color statistics and directional texture cues. Based on CGBlock, we further construct a fusion block named C2CG and insert it into three key stages to enhance semantic region modeling. The overall structures of C2CG and CGBlock are shown in Figure 6.

CGBlock consists of two parallel attention branches.

In the color-aware branch, we extract the Hue (H) and Saturation (S) components from the HSV transformation of the input image. These components are adaptively averaged using Global Adaptive Average Pooling. The two global descriptors are element-wise multiplied to form a joint color representation, which is then passed through a two-layer MLP consisting of a Fully Connected (FC) layer, a ReLU activation, a second FC layer, and a final Sigmoid activation to obtain the color attention weights:

(25) $$\gamma_{c}=\sigma \left(W_{2}\cdot \delta \left(W_{1}\left(H⊙S\right)\right)\right),$$

where $W_{1}\in R^{C^{\prime }\times C}$ and $W_{2}\in R^{C\times C^{\prime }}$ are the learnable weights of two fully connected layers, δ(·) denotes the ReLU activation, σ(·) denotes the Sigmoid activation, and ⊙ represents element-wise multiplication between the global average pooled Hue (*H*) and Saturation (*S*) vectors. The resulting attention vector $\gamma_{c}\in R^{C}$ is used to modulate the input features based on color priors.

In the texture-aware branch, the input feature *F* is convolved with a set of Gabor filters $G^{\left(\theta_{k}\right)}$ at predefined orientations $\theta_{k}$. The resulting directional responses are concatenated along the channel dimension:

(26) $$R=\mathrm{Concat}\left(R_{1},R_{2},...,R_{K}\right)\in R^{KC\times H\times W},$$

where $R_{k}=G^{\left(\theta_{k}\right)}⊙F$ represents the response at orientation $\theta_{k}$.

The concatenated response is normalized using GroupNorm and projected back to the original channel dimension *C* using a 1×1 convolution, followed by a Sigmoid activation to produce the texture attention weights:

(27) $$\gamma_{g}=\sigma \left({\mathrm{Conv}}_{1\times 1}\left(\mathrm{GN}\left(R\right)\right)\right).$$

The two attention weights $\gamma_{c}$ and $\gamma_{g}$ are separately applied to modulate the input feature *F* via channel-wise multiplication. The original feature *F*, the color-modulated feature $\gamma_{c}⊙F$, and the texture-modulated feature $\gamma_{g}⊙F$ are then concatenated and compressed through a 1×1 convolution to produce the final output:

(28) $$F_{\mathrm{out}}={\mathrm{Conv}}_{1\times 1}\left(F\|\left(\gamma_{c}⊙F\right)\|\left(\gamma_{g}⊙F\right)\right),$$

where ‖ denotes channel-wise concatenation.

To incorporate CGBlock into the overall detection framework, we build the C2CG module. The C2CG module adopts the same structural design as the original C2PSA module in YOLOv11 but replaces its internal PSABlock with two sequential CGBlocks.

Specifically, the input feature first passes through a CBS module, followed by a CGBlock. A shortcut connection is established, and the output is processed by another CGBlock. The two outputs are then concatenated along the channel dimension and fused by a final CBS layer. This structure enhances feature expressiveness by integrating color and texture priors at multiple stages.

We insert the C2CG modules at three critical stages of the network: immediately after the backbone to bridge it with the neck, within the intermediate fusion layers of the neck, and directly preceding the final detection heads. At each location, the C2CG module serves as a dual-path attention enhancer that adaptively refines semantic features, thereby improving the localization of regions with prominent color or texture cues.

By explicitly injecting color-guided and direction-sensitive attention into intermediate features, our CGBlock and C2CG modules enable the network to better capture the perceptually salient and culturally symbolic elements in Thangka paintings, such as facial regions, ornaments, and aura outlines. As a result, our model achieves higher confidence and better spatial precision in detecting fine-grained targets.

### 3.4. Multi-Scale Detection Head Enhancement

YOLOv11 uses a three-scale detection head to manage objects of different sizes. However, Thangka images are especially difficult because they contain ultra-small, densely packed targets such as ritual implements, lotus bases, and golden edge ornaments. These objects often cover only a few pixels and are hard to distinguish in complex scenes.

To improve detection of such fine-grained structures, we propose a dual enhancement strategy: first, we redesign all three original detection heads to be more expressive and modular; second, we introduce an additional shallow detection head at the early stage of the neck. This head is inserted immediately after the first LLWB_Up and fusion step, where high-resolution features retain fine spatial detail, enabling improved localization of small and dense targets.

**Figure 7:**
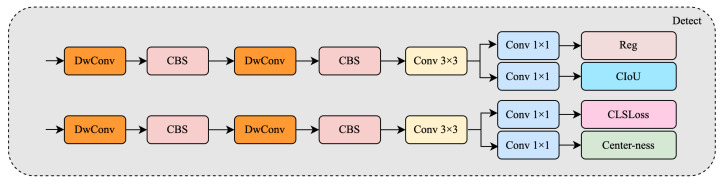
Structure of the improved detection head. The head adopts lightweight depthwise separable convolutions and introduces a center-ness prediction branch alongside the classification loss, enabling better localization of small or dense objects. Each branch outputs prediction features through a Conv 3×3 followed by parallel Conv 1×1 layers for regression, CIoU, classification, and center confidence.

Figure 7 shows a structural comparison between the original YOLOv11 detection head (bottom) and our improved head (top). While the original head employs a shallow layout combining CBS and depthwise convolution paths, our improved version deepens the structure by stacking two depthwise separable convolution blocks, each followed by a CBS module. This is followed by a 3×3 convolution and multiple 1×1 convolutional branches to output the final predictions.

Notably, our classification branch is extended with a center-ness prediction head, inspired by anchor-free detectors such as FCOS [7]. This additional output improves the network’s ability to localize the most confident object centers, which is especially beneficial for small-scale or visually ambiguous targets. Furthermore, regression and IoU confidence prediction are explicitly decoupled into parallel 1×1 convolutions, providing clearer gradient paths and better convergence.

Beyond structural refinement, we introduce an additional detection head that branches from a shallow stage in the neck. This head processes higher-resolution features and complements the three original scales, focusing specifically on small targets that may be missed by deeper layers. All four heads share the same improved structure and are trained jointly.

During inference, predictions from all four detection heads are aggregated, forming a unified multi-scale output. This enhanced head design significantly improves mAP on small objects and boosts recall in dense scenarios, while maintaining inference efficiency and architectural consistency with YOLOv11.

## 4. Experiments

### 4.1. Experimental Setup

We adopt standard object detection metrics to evaluate model performance, including mean Average Precision (mAP) at various IoU thresholds and recall. The mAP is defined as:

(29) $$\mathrm{mAP}@t=\frac{1}{C}\sum_{c=1}^{C}\int_{0}^{1}{\mathrm{Precision}}_{c}\left(p\right)\;dp,$$

where *t* denotes the IoU threshold, *C* is the number of object classes, and ${\mathrm{Precision}}_{c}\left(p\right)$ is the precision–recall curve for class *c*. We report both mAP@0.5 and mAP@[0.5:0.95], where the latter is the average of results over ten IoU thresholds ranging from 0.5 to 0.95 in steps of 0.05.

Recall is defined as:

(30) $$\mathrm{Recall}=\frac{\mathrm{TP}}{\mathrm{TP}+\mathrm{FN}}$$

where TP and FN represent the number of true positives and false negatives, respectively. Recall reflects the completeness of detection, which is particularly important in densely annotated scenes.

All models are implemented using the PyTorch v1.13.1 framework and trained on a single NVIDIA RTX 3080 Ti GPU (NVIDIA Corporation, Santa Clara, CA, USA). Input images are resized to 640×640. The AdamW optimizer is used with an initial learning rate of 0.0005, a batch size of 32, and 300 training epochs. The learning rate follows a cosine annealing schedule, with a linear warm-up during the first 10 epochs.

All models are initialized from ImageNet pre-trained weights. The proposed modules introduced in this work, including LLWB and CGBlock, are trained from scratch using Kaiming initialization, which is well-suited for ReLU-based activations.

During both training and evaluation, multi-scale testing and Test-Time Augmentation (TTA) are disabled to ensure fair and consistent comparisons across models.

### 4.2. Dataset and Augmentation

We construct a Thangka object detection dataset using high-resolution images collected from publicly available digital sources and archival repositories. The dataset includes artworks from diverse artistic styles and materials such as canvas, silk, and paper-based Thangka. All images are annotated in YOLO format by domain experts, covering 10 fine-grained object categories: Main Figure, Side Deity, Halo, Crown, Ornament, Weapon, Flame, Lotus Base, Animal, and Plant. These categories were defined by Thangka experts based on their semantic distinctiveness and visual recurrence. In total, the dataset comprises approximately 3747 images, with an average of 3–5 labeled instances per image.

During the data collection process, we also included a number of historical Thangka artworks, many of which exhibit real-world degradation such as pigment erosion, visible stains, physical damage, or blur due to aging and preservation artifacts. To enhance the robustness of our detection model in such complex and noisy scenarios, we design augmentation strategies that intentionally simulate these conditions.

The augmentation pipeline includes random grayscale conversion, color jittering, occlusion masking, random cropping, horizontal flipping, Gaussian blur, and rotation. These augmentations aim to imitate degradation patterns and sensor noise. In addition, to increase the model’s adaptability to different base-color schemes, we apply a CycleGAN [39] style transfer to convert colorful Thangka backgrounds into black, red, blue, and other variants. We further adopt a miniature reproduction strategy, in which a scaled-down version of the original image is overlaid back onto itself, enriching the spatial hierarchy and facilitating foreground–background separation.

The augmented dataset is used consistently for all training configurations to ensure fair and reproducible evaluation.

### 4.3. Overall Performance Comparison

To comprehensively evaluate the effectiveness of our proposed enhancements, we compare our model against a range of representative object detectors, including Faster R-CNN, SSD, DETR, YOLOv5, YOLOv8, YOLOv10, and YOLOv11. These models cover both classical two-stage detectors and modern one-stage detectors with diverse architectural designs. Faster R-CNN and SSD represent traditional two-stage and early single-stage frameworks, respectively. DETR introduces transformer-based end-to-end detection without region proposals. YOLOv5 and YOLOv8 are popular lightweight detectors balancing speed and accuracy, while YOLOv11 bring further structural optimizations and attention mechanisms. Our method builds upon YOLOv11 and incorporates frequency-aware modeling, prior-guided attention, and shallow head enhancements to better address the unique challenges of Thangka image detection.

All models are trained and evaluated under identical conditions using our Thangka dataset and the same set of augmentation strategies. We report mAP@0.5, mAP@[0.5:0.95], recall, and model size in terms of parameter count, as shown in Table 1.

From the results, we observe that our enhanced YOLOv11 model consistently outperforms all baseline methods across all reported metrics. Specifically, our model achieves 89.5% mAP@0.5, 59.4% mAP@[0.5:0.95], and 84.7% recall, significantly surpassing the performance of Faster R-CNN (78.5% mAP@0.5, 48.9% mAP@[0.5:0.95], 80.3% recall), SSD, DETR, YOLOv5, and YOLOv8. Compared to the original YOLOv11 baseline, our approach improves mAP@0.5 by 5.6 points and recall by 6.5 points, demonstrating the strong impact of the proposed frequency-aware, prior-guided, and shallow detection head enhancements. Furthermore, despite the significant performance gains, our model remains lightweight, with only 20.9 M parameters, close to YOLOv11’s 20.1 M, indicating an excellent trade-off between accuracy and efficiency. These results validate the effectiveness and generalizability of our design in detecting small-scale, densely packed, and richly textured Thangka elements.

These results validate the effectiveness of the proposed modules. The LLWB-based frequency modeling improves the structural alignment of features, especially in complex textured regions. The C2CG attention module further reinforces localization by emphasizing chromatic and directional cues. Additionally, the added shallow detection head contributes to the recall of fine-grained objects such as small ornaments and decorative patterns, which are often missed by deeper detection layers alone.

**Figure 8:**
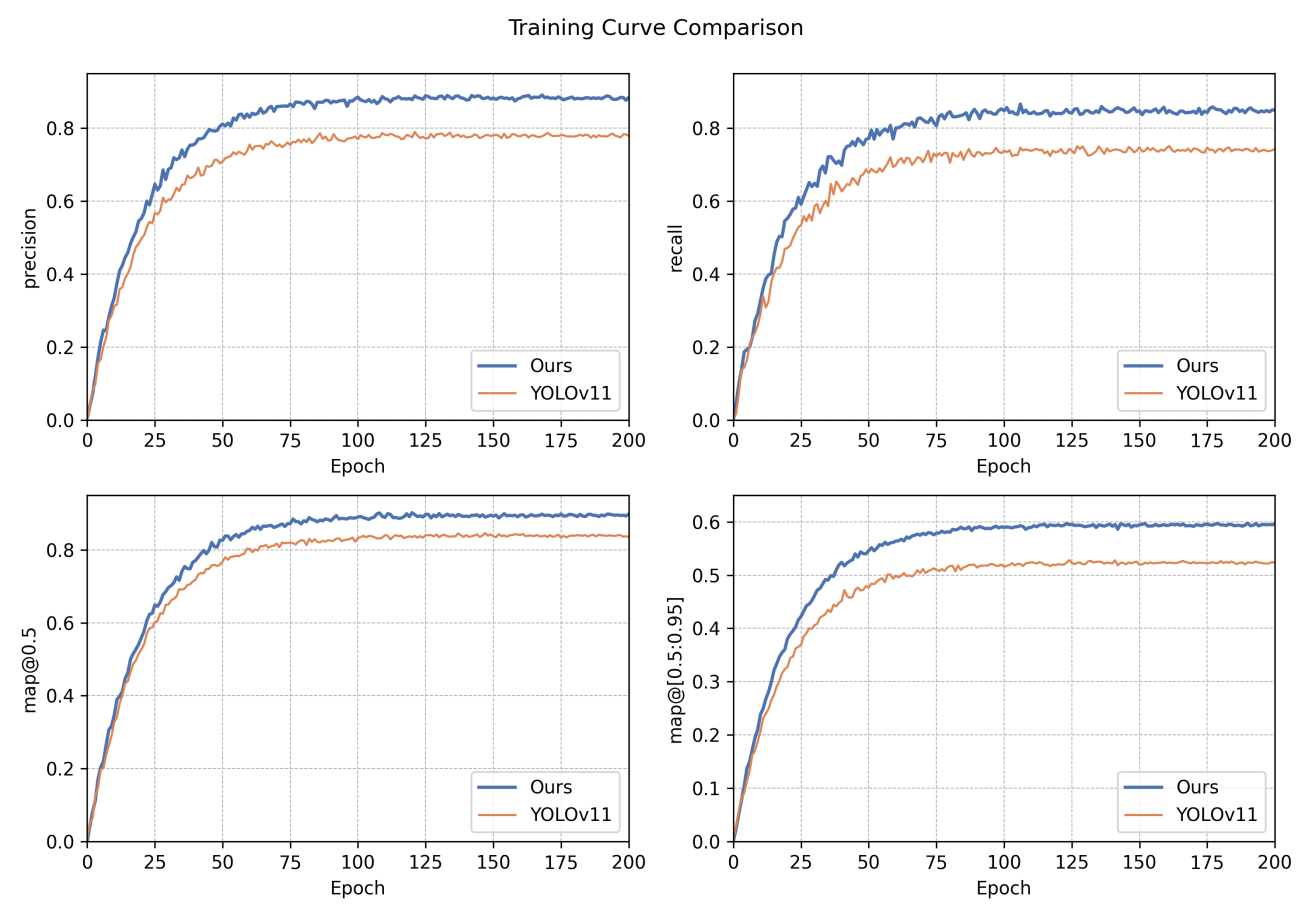
Training curve comparison between our enhanced YOLOv11 model and the original YOLOv11 baseline. Our model achieves faster convergence and higher stability across all metrics: precision, recall, mAP@0.5, and mAP@[0.5:0.95].

To further validate the convergence behavior and training stability of our proposed model, we compare its training dynamics with the YOLOv11 baseline. As shown in Figure 8, our model exhibits faster convergence and consistently higher values in all key metrics, including precision, recall, mAP@0.5, and mAP@[0.5:0.95]. This demonstrates that our modifications not only improve final performance but also enhance optimization stability during training.

Overall, our method demonstrates strong generalization ability and robustness in detecting multi-scale, richly styled targets in Thangka imagery, outperforming both conventional and Transformer-based approaches.

### 4.4. Ablation Study

To evaluate the effectiveness and interaction of the proposed modules, we conduct a series of ablation experiments under consistent training conditions. We examine the individual impact and combined effects of the three key components: the frequency modeling module (LLWB Series), the prior-guided attention module (C2CG), and the shallow detection head (Head). The LLWB Series includes the LLWB for backbone enhancement and the LLWB_Down and LLWB_Up modules for frequency-guided multi-scale feature fusion. This grouping better reflects the holistic integration of frequency-aware modeling across both the backbone and neck stages. Each configuration is trained from scratch using the same dataset and augmentation strategies. The results are summarized in Table 2.

As shown in Table 2, each module individually contributes to the model’s performance. When LLWB is applied alone, mAP@0.5 increases by 2.8%, indicating that frequency decomposition enhances the model’s ability to encode structural and texture cues. The C2CG module improves mAP@[0.5:0.95], suggesting that attention guided by color and orientation helps the model focus on semantically salient regions. We also redesigned all detection heads with decoupled convolutional branches and added an additional shallow head. This change increases the recall rate by 3.3% and helps the model localize small-scale and peripheral targets more effectively.

The combinations of modules show clear synergy. When LLWB and C2CG are combined, both precision and recall improve, confirming their complementary roles in semantic modeling. The LLWB + Head configuration also improves recall, indicating that frequency-guided features enhance small-object localization. The C2CG + Head combination yields modest mAP gains but further increases recall, suggesting that salient region guidance enhances multi-scale robustness.

Finally, enabling all three modules produces the best results across all metrics, achieving 89.5% mAP@0.5, 59.4% mAP@[0.5:0.95], and 84.7% recall. While this configuration incurs a slight speed reduction from 56 FPS to 52 FPS, it demonstrates that the proposed components offer substantial accuracy gains with only minor computational overhead—yielding an effective trade-off for real-world deployment in Thangka image analysis.

**Figure 9:**
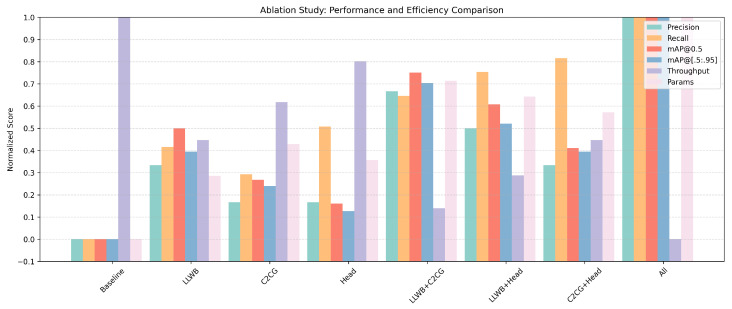
Normalized comparison of different module combinations in terms of performance and efficiency. Each metric is scaled to [0,1], with the best configuration assigned 1 and the worst 0. The metrics include precision, recall, mAP@0.5, mAP@[0.5:0.95], throughput, and parameter size. This figure complements Table 2 by highlighting the trade-off between accuracy and efficiency.

To better visualize the performance–efficiency trade-offs introduced by each component, we present a normalized bar chart in Figure 9, covering accuracy-related metrics (precision, recall, mAP), efficiency metrics (throughput), and model size (parameters). Each metric is normalized between 0 and 1, where higher is better, except for model size (normalized inversely). From this visualization, it is evident that the full model achieves the best overall balance across all dimensions.

### 4.5. Cross-Validation Results

To further assess the generalization ability and robustness of our proposed method, we conducted 5-fold cross-validation on the entire Thangka detection dataset. In each fold, 80% of the data was used for training and 20% for validation, ensuring that each sample was evaluated exactly once as part of the validation set. Table 3 reports the mAP@0.5, mAP@[0.5:0.95], and recall for each fold, as well as the overall mean and standard deviation across folds.

The results demonstrate that our model achieves consistently high detection performance across different data splits, with only minor variance. This confirms the stability and strong generalization ability of our approach, reducing the risk of reporting results due to a particularly favorable split.

### 4.6. Visualization Analysis

To further interpret the behavior and effectiveness of our proposed detector, we provide three types of visualizations: (1) sample detection results on diverse Thangka images, (2) Grad-CAM-based attention heatmaps, and (3) the confusion matrix of the proposed model.

**Figure 10:**
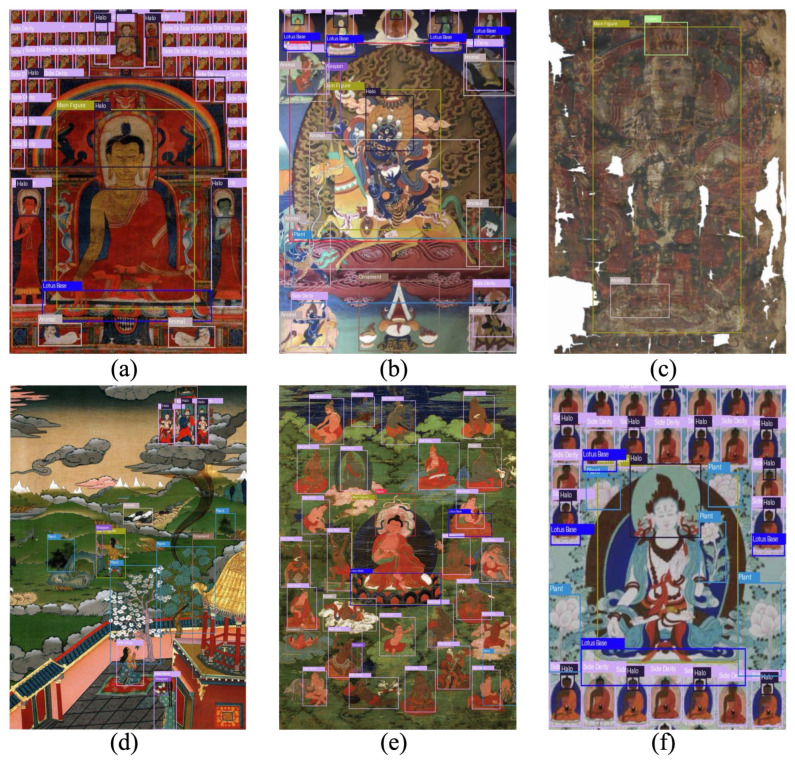
Detection results on representative Thangka images: (**a**) single-color background Thangka, (**b**) multi-class richly decorated image, (**c**) severely damaged image, (**d**) narrative scene, (**e**) crowded character scene, (**f**) repeated deity structure. The model successfully localizes small and occluded targets while adapting to different layout complexities.

As shown in Figure 10, our model demonstrates robust generalization across various types of Thangka. It detects small, densely packed components (e.g., Side Deity, Halo) in cluttered layouts and still performs well under challenging conditions such as damaged artworks (c) or high-density narratives (e). The model is also able to distinguish repetitive structures and similar visual patterns, validating the contribution of frequency-aware and prior-guided enhancements.

**Figure 11:**
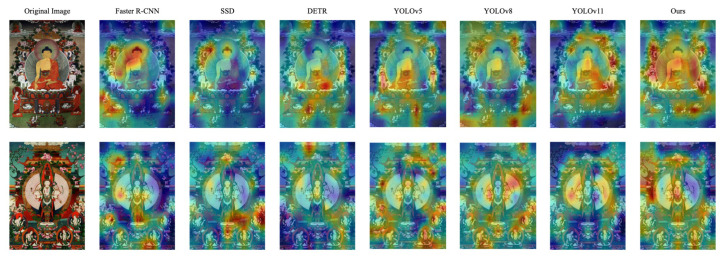
Grad-CAM visualizations comparing the attention regions of different detectors. Our model exhibits more focused activation on the target figure regions, particularly on Main Figure and key symbolic parts such as Halo and Lotus Base.

Figure 11 illustrates the attention distribution of different models using Grad-CAM [40]. Compared with other methods, our approach consistently concentrates on semantically meaningful regions. In both examples, it produces more interpretable and spatially precise attention, indicating better semantic grounding and alignment with human perception.

**Figure 12:**
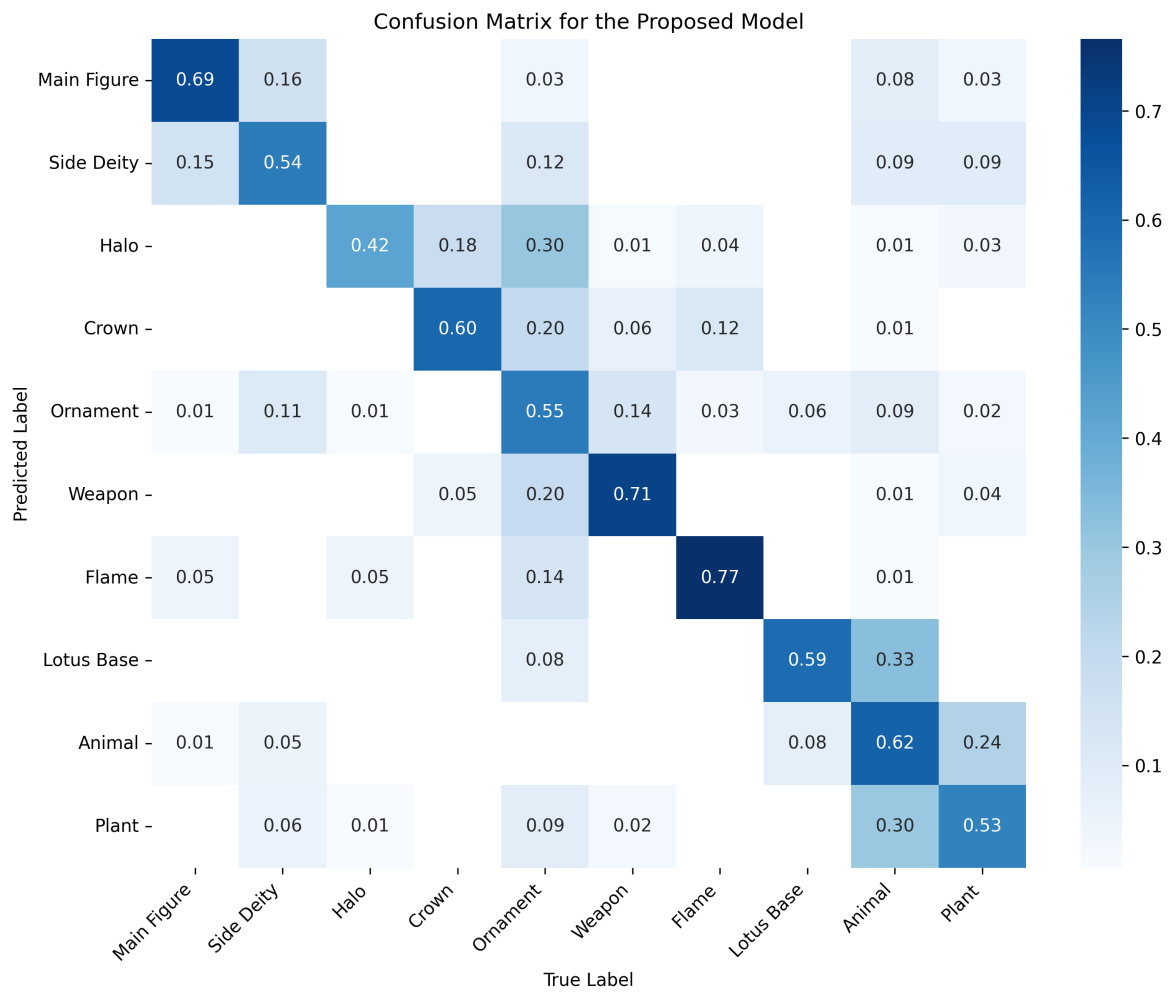
Confusion matrix of our model. The diagonal dominance indicates high overall accuracy, while some confusion remains between visually similar categories such as Halo vs. Crown and Animal vs. Plant.

Figure 12 visualizes the confusion matrix for our model across all 10 object categories. The diagonal dominance indicates generally strong classification performance, although some misclassification is observed between visually or semantically similar classes such as Crown and Halo or Animal and Plant.

To provide a more quantitative and category-specific evaluation, Table 4 presents the per-class precision, recall, and F1-score computed from the confusion matrix. These metrics reveal both the strengths and the areas for improvement in each category, highlighting that the model achieves balanced performance across most major categories, while small or visually ambiguous classes (e.g., Ornament, Animal, Plant) remain more challenging.

These visualizations and metrics collectively confirm that our model not only achieves strong overall detection performance but also learns discriminative features for most categories in the complex Thangka domain.

## 5. Discussion

In this paper, we propose a frequency- and prior-enhanced object detection framework tailored to the unique characteristics of Thangka imagery. Our approach builds upon the YOLOv11 backbone and introduces three key innovations: a LLWB for frequency-aware feature decomposition, a C2CG for enhancing semantic focus, and a redesigned detection head structure that integrates center-aware confidence and lightweight convolutions. Furthermore, we introduce an additional shallow detection head at the early stage of the neck to improve the recall of ultra-small and densely packed objects.

Through extensive experiments on a carefully curated Thangka dataset, our method achieves consistent improvements over both conventional CNN-based detectors and recent Transformer-based architectures. Notably, we observe significant gains in mAP@0.5, mAP@[0.5:0.95], and recall, while maintaining a relatively compact model size. Ablation studies further validate the individual effectiveness and complementary synergy of each proposed module.

The proposed framework demonstrates strong capability in detecting complex, multi-scale, and richly ornamented objects under challenging visual conditions such as color variance, texture clutter, and background noise. While our design is tailored for Thangka imagery, it remains to be seen how well the method generalizes to other forms of cultural heritage with distinct visual styles (e.g., murals, manuscripts). In particular, heavily occluded or degraded components still pose challenges that are not fully resolved by the current modules. Moreover, the framework assumes an offline detection setting and does not yet meet the computational constraints of real-time deployment. Despite these limitations, the modular enhancements we propose are potentially transferable to other domains. In future work, we plan to explore these extensions and incorporate symbolic or textual attributes for more comprehensive cultural interpretation.

## Figures and Tables

**Table 1:** Performance comparison with state-of-the-art detectors.

Model	mAP@0.5	mAP@[0.5:0.95]	Recall	Params (M)
Faster R-CNN	78.5	48.9	80.3	28.5
SSD	80.1	49.8	76.2	26.3
DETR	82.4	51.5	77.0	36.8
YOLOv5	82.0	50.8	76.9	21.2
YOLOv8	82.8	51.5	77.5	25.9
YOLOv11	83.9	52.3	78.2	**20.1**
**Ours**	**89.5**	**59.4**	**84.7**	20.9

**Table 2:** Ablation study on the proposed modules (FPS measured/estimated on RTX 3080 Ti @640 × 640, batch = 1, FP32). ↑ indicates that higher values are better. √ denotes that the corresponding module is enabled. Bold values highlight the best results.

LLWB Series	C2CG	Head	Accuracy			FPS ↑
			mAP@0.5	mAP@[0.5:0.95]	Recall	
			83.9	52.3	78.2	**56**
√			86.7	55.1	80.9	54
	√		85.4	54.0	80.1	55
		√	84.8	53.2	81.5	55
√	√		88.1	57.3	82.4	53
√		√	87.3	56.0	83.1	53
	√	√	86.2	55.1	83.5	54
√	√	√	**89.5**	**59.4**	**84.7**	52

**Table 3:** Five-fold cross-validation results for the proposed model (mean ± std).

Fold	mAP@0.5	mAP@[0.5:0.95]	Recall
Fold 1	89.5	59.1	84.4
Fold 2	89.8	59.4	84.7
Fold 3	89.2	59.3	84.5
Fold 4	89.7	59.6	84.8
Fold 5	89.4	59.5	84.6
Mean ± Std	89.5 ± 0.2	59.4 ± 0.2	84.6 ± 0.1

**Table 4:** Per-class precision, recall, and F1-score for the proposed model.

Class	Precision	Recall	F1-Score
Main Figure	0.80	0.70	0.75
Side Deity	0.56	0.55	0.55
Halo	0.71	0.42	0.53
Crown	0.68	0.61	0.64
Ornament	0.37	0.54	0.44
Weapon	0.76	0.70	0.73
Flame	0.74	0.75	0.75
Lotus Base	0.64	0.59	0.61
Animal	0.46	0.62	0.53
Plant	0.53	0.52	0.53

## Data Availability

The datasets generated and/or analysed during the current study are not publicly available due to concerns related to cultural sensitivity and image authorization agreements. However, the data are available from the corresponding author upon reasonable request.

## Abbreviations

The following abbreviations are used in this manuscript:

LLWB	Learnable Lifting Wavelet Block
CGBlock	Color-Gabor Block
C2CG	Color-Gabor Cross Gate
RPN	Region Proposal Network
SPFF	Spatial Pyramid Feature Fusion
C2PSA	Cross-Stage Partial Spatial Attention
CBS	Convolution-BatchNorm-SiLU
QWT	Quaternionic Wavelet Transform
WBCT	Wavelet based Contourlet Transformer
DWCM	Dual-Wavelet Convolution Modules
AGCNs	Adaptive Gabor Convolutional Networks
SAR	Synthetic Aperture Radar
DWT	Discrete Wavelet Transform
CBCA	Cross-Band Channel Attention
LL-DWT	Lifting Discrete Wavelet Transform
LL-IWT	Lifting Inverse Wavelet Transform
TTA	Test-Time Augmentation
